# Assessing the freshwater flux from the continents to the Mediterranean Sea

**DOI:** 10.1038/s41598-019-44293-1

**Published:** 2019-05-29

**Authors:** Fuxing Wang, Jan Polcher

**Affiliations:** Laboratoire de Météorologie Dynamique, IPSL, CNRS, Ecole Polytechnique, 91128 Palaiseau, France

**Keywords:** Climate and Earth system modelling, Hydrology, Hydrology, Natural hazards, Scientific data

## Abstract

Precipitation minus evaporation over continents is the freshwater flux which can be sustainably exploited by mankind. Over a catchment and longer time periods, this flux is also the amount of water which flows into the ocean. An essential question for semi-arid areas of the world is how well this freshwater flux can be estimated and predicted to evolve under climate change and human water use. Knowing this flux is thus an essential element in regional water resources management. Here we examine this question over the catchment of the Mediterranean Sea. Using a novel data assimilation methodology that incorporates observed discharges of rivers in a land surface model, new estimates of the freshwater flux to the Mediterranean Sea for the period 1980–2013 are proposed. We find that more freshwater (40–60%) flows into the sea than previously estimated. The hypothesis we advance is that previous estimates have underestimated the discharges of the large number of unmonitored coastal basins and neglected submarine ground water flows. The proposed error bars on the estimate indicate that the degrading river gauging station network limits our ability to monitor this branch of the water cycle reliably. Nevertheless, the uncertainty is small enough to allow the identification of regions in which non-climatic decreases in the freshwater flows exist over the period.

## Introduction

The Mediterranean region is considered to be one of the most vulnerable regions to climate change as water scarcity is expected to be exacerbated^1,2^. The renewable water resources are predicted to decrease with climate change as a result of increasing temperature and reduced rainfall^3^. These changes are particularly important for this region with already scarce water resources and increasing demands for water for domestic, industrial, irrigation, and tourism activities^4^. According to the United Nations World Water Development Report^5^, the Mediterranean is a region which includes catchments where water consumption exceeds the locally renewable water resources by a factor of two.

The renewable water resources can be characterised by the flow from the continents to the oceans, as it is the residual of the water exchanges between the continent and the atmosphere. Water discharge from the continents also plays an important role for the Mediterranean Sea as it provides a large fraction of the freshwater^6^ and most nutrients^7^. Because of the semi-enclosed nature of this sea, these fluxes drive in large part the oceanic circulation patterns and the marine productivity^8^. The impact of climate change and human water usage on the quantity and quality of the flux from the continents will induce changes in salinity, thermohaline circulations^9,10^, biological productivity and the ecological state of the sea^11,12^.

**Figure 1:**
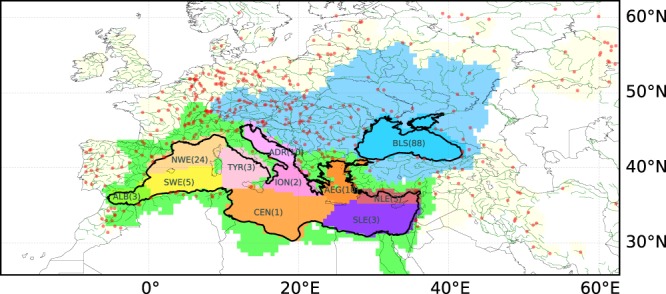
Map of hydrological catchment of the Mediterranean (green) and the Black sea (blue) and the GRDC stations which have been assimilated (red dots) into ORCHIDEE. The sub-basins of the sea are labelled as follows: Alboran (ALB), South-Western (SWE), North-Western (NWE), Tyrrhenian (TYR), Adriatic (ADR), Ionian (ION), Central (CEN), Aegean (AEG), North-Levantine (NLE), South-Levantine (SLE) and Black Sea (BLS). For each sub-basin the number of GRDC stations assimilated on its tributaries is provided in brackets.

Thus the quantification of the freshwater flux with its space and time variability will help monitor water availability over the continental catchments and the main drivers of the marine productivity. Previous attempts at estimating this flux were at low spatial and temporal resolutions^13,14^ because they were largely based on observations from large gauged rivers. The complex morphology of the Mediterranean Sea with sharp orographic features and a tortuous coastline (Fig. 1)^15^ lead to a large number of small unmonitored catchments which are not well taken into account in these estimates. Furthermore, the Mediterranean is one of the regions of the world with the most karstic submarine and brackish coastal springs along the coasts^16,17^ which are not considered in previous quantifications of the freshwater flux. Considering the importance of the freshwater flux into the Mediterranean to our society, our current knowledge of this essential flux is insufficient.

In this study, we estimate riverine discharge through the Fusion of ORCHIDEE (Organising Carbon and Hydrology In Dynamic Ecosystems)^18,19^ land surface model (LSM) and Global Runoff Data Center (GRDC, 56068 Koblenz, Germany) observations over the Mediterranean catchments for the 1980–2013 period (called hereafter FOG: Fusion of ORCHIDEE and GRDC). FOG corrects modelling and forcing errors in the continental moisture convergence and thus also provides adjustments for unmonitored catchments.

## Comparison of this New Freshwater Estimation with Previous Studies

The methodologies to estimate the freshwater flux from continents to the oceans can be classified into two large categories which can also be combined: (i) observed river discharge based (ODB) methods and (ii) moisture convergence based (MCB) methods. The strength of ODB is that it is based on one of the most reliable observations of the continental water cycle while the power of the MCB method is that it covers also unmonitored basins and implicitly the groundwater fluxes to the ocean. This also means that ODB methods are limited in regions with complex coastlines where the contribution of small unmonitored of submarine groundwater discharge (SGD) is important. While MCB methods are limited by poor precipitation observations or in regions where evaporation is not well estimated. For the fluxes into the Mediterranean (MED) this is in particular the case for the Nile basin where evaporation is underestimated^20^ leading to overestimated discharge for this river (See section Method).For the Mediterranean area we also have estimates which are based on national water statistics, which is probably a unique situation especially since they provide also some information on SGD.

### Freshwater flux estimation by FOG

**Figure 2:**
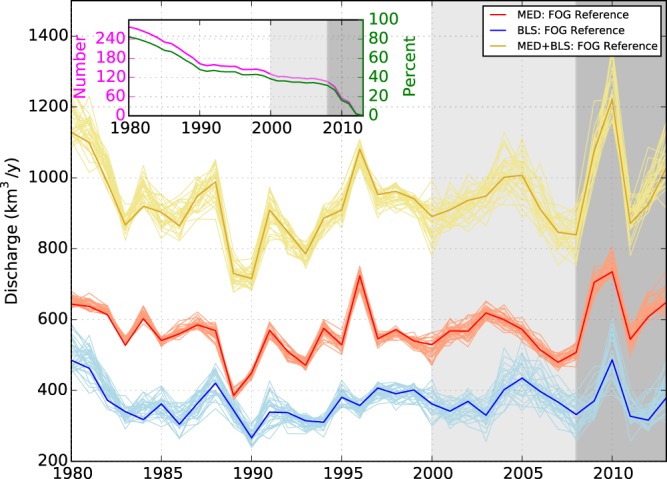
Evolution of total discharge into the Mediterranean Sea (red line), the Black sea (blue line) and the total over both seas (gold lines) over 1980–2013 together with the error range from 30 ensemble members (light colour lines). The top left figure shows the number (purple) and percentage (green) of GRDC stations used in FOG over time. The grey and dark grey shaded area indicates the period where the percentage of GRDC stations are lower than 40% (2000 to 2008) and lower than 30% (2009 to 2013), respectively.

The freshwater inputs into the Mediterranean Sea and the Black Sea are estimated to be 569 ± 149 *km*^3^/*y* and 367 ± 61 *km*^3^/*y*, respectively over the 1980–2013 period (Table 1). The ORCHIDEE LSM reference simulation shows higher discharge values, but which are still within the uncertainty range of FOG, probably because the model underestimates evaporation in this region. The fusion process corrects moisture convergence by adjusting evaporation to compensate for errors in the forcing or missing processes (irrigation, reservoirs and dams, and floodplains) in ORCHIDEE^21^, thus correcting some of the issues of MCB methods with the strength of ODB methods. The proposed uncertainty range explores the space opened by the residual error at gauging stations when observations are available, inter-annual variability when climatology is used, and the extrapolation of the correction factor. This covers a broad range of error sources. As can be seen by the widening of the ensemble toward the end of the period (Fig. 2), the main driver is the decline of the observational network and the missing observations after 2012.

**Table 1:** Literature review of water fluxes into the Black Sea and Mediterranean.

Source	Discharge [*km*^3^/*y*]			Period	Notes
	BLS	MED	Total		
Kara *et al*.^22^	287			1952–1984	ODB
Jaoshvili *et al*.^23^	294 to 474			Climatology	Literature review
Boukthir and Barnier^46^		347		1974–1994	ODB
Mariotti *et al*.^47^ Struglia *et al*.^48^		256 to 328		Climatology	ODB
Peucker-Ehrenbrink^49^	414	561	975	Climatology	ODB
Margat and Treyer^24^		473.5		Climatology	Based on national water statistics. 43.5 *km*^3^/*y* of the discharge are attributed to SGD
Bouraoui *et al*.^8^		282 to 327		1980–2000	MCB without the Nile
Fekete *et al*.^26^			869	Climatology	ODB/MCB combination without the Nile.
Dai and Trenberth^27^ (Largest 921)			838	Climatology	ODB
Dai and Trenberth^27^ (Fekete runoff)			864	Climatology	MCB without the Nile.
Dai and Trenberth^27^ (re-analaysis P-E)			409–538	Climatology	MCB without the Nile.
Syvitski *et al*.^50^			710	Climatology	ODB/MCB combination
aus der Beek *et al*.^20^	406	318	724	2002–2009	MCB without the Nile.
Szczypta *et al*.^28^	451	317	768	1991–2008	MCB without the Nile.
Ludwig *et al*. (CEFREM-LR)^14^	387	345	727	1960–2000	ODB/MCB combination
Ludwig *et al*. (CEFREM-HR)	400	398	798	1980–2009	ODB/MCB combination
This study: ORCHIDEE	389	575	964	1980–2013	MCB
This study: FOG	367 ± 61	569 ± 149	936 ± 210	1980–2013	MCB with assimilation of observed discharge

In the notes to each estimate we distinguish between observed discharge based (ODB) or moisture convergence based (MCB) methods and other approaches to estimate the discharge.

### Comparison of freshwater flux by FOG with previous estimates

Comparing FOG to previous estimates for the Black Sea (BLS in Table 1) shows good agreement, especially with Centre de Formation et de Recherche sur les Environnements Méditerranéens’ (CEFREM’s) estimates^14^, which have been used extensively for oceanic modelling and water balance studies^6^. This can be explained by the fact that a few large and well-gauged rivers dominate the total fluxes in both datasets. The estimate by Kara *et al*.^22^ is lower, but the range proposed by Jaoshvili *et al*.^23^ is larger than the uncertainty of FOG. The difference in the periods considered can probably explain this.

**Figure 3:**
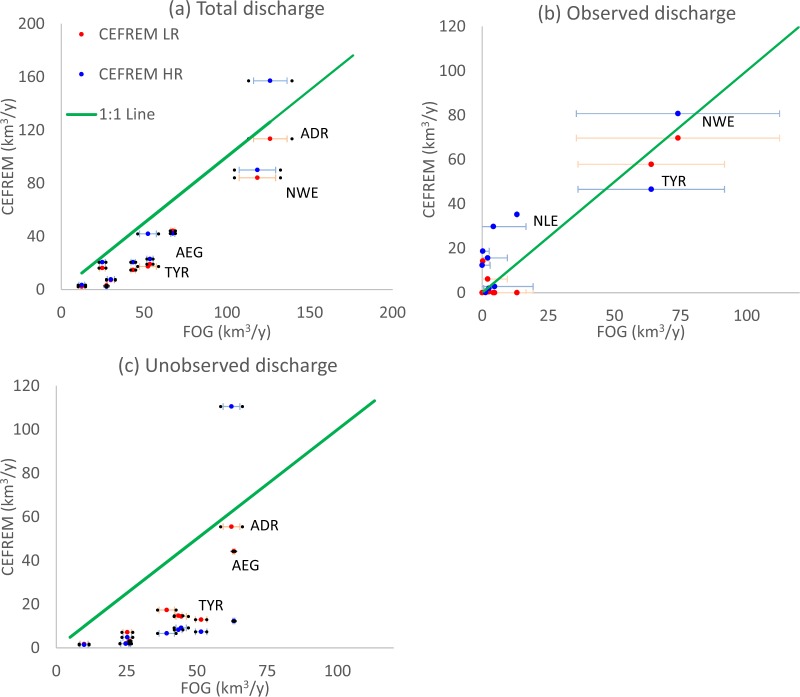
(**a**) The scatter plot for total discharge FOG versus of CEFREM (LR in red, HR in blue) over 10 oceanic basins (represented by dot). The negative and positive error lines (both in blue and red) represent the average of 5 and 95 percentiles. The black dots on the left and right side of the error lines show the minimum and maximum values of the 30 ensembles, respectively. (**b**) As previous figure but only including coastal points to which gauged rivers lead and with the uncertainty represented by residual errors. **(c**) as (**b**) but for catchments with unmonitored catchments. Values are computed for the overlap period 1980–2000.

For the MED, FOG has higher values than all previous estimates (MED in Table 1), except when compared to the values derived from national water statistics and which include SGD^24^. Compared to the CEFREM estimate, FOG suggests that 65% (Low Resolution of CEFREM) or 43% (High Resolution of CEFREM) more freshwater flows into the Mediterranean for the overlap period (1980–2000).This difference is larger than the error margin associated to the FOG estimate. For basins, such as ADR or NWE, which are dominated by Alpine catchments, the difference to CEFREM is below 40% (Figs 3a and S1). Over the ADR basin, the CEFREM-LR is within the error bar of FOG. The flux estimated here is up to 2–4 times larger for ALB, SWE, TYR, ION, CEN, and NLE (Figs 3a and S1).

Previous studies^14,22,23^ indicated that the freshwater discharge into the Black Sea is greater than that discharged into the Mediterranean Sea (Table 1). Our results suggest that the opposite is the case, in particular because of higher fluxes proposed by FOG in the north eastern part of the Mediterranean Sea. This result is essential for salt budget analysis and modelling studies of the Mediterranean Sea because using the proposed freshwater inflow will lead to less saline surface waters in basins, such as the Aegean and Northern Leventine, which can impact the formation of the Leventine Intermediate Water and the exchanges through the Turkish straight system^25^. On the other hand, when the combination of BLS and MED is considered most other studies are in the lower uncertainty range of FOG, whether they are ODB, MCB or a combination of both methods (Table 1). If the Nile is included, then most estimates will be higher than FOG because of the large discharges obtained for this river^20,26–28^.

### The origin of the difference between FOG and previous works

To better understand the difference with CEFREM, discharge for the different oceanic basins is separated into values from coastal points with observations (in CEFREM-LR) and unmonitored catchments. The coastal point constrained by observations in FOG, CEFREM-LR, and CEFREM-HR mostly are within our uncertainty (Fig. 3b). However, for unobserved coastal points, the river discharges are higher than CEFREM-LR and CEFREM-HR over most Mediterranean basins (Fig. 3c). This indicates that the higher values of FOG compared to CEFREM can be explained mainly by the divergence on coastal points without gauged catchments.

### The role of physically-based LSM in un-monitored river basins

In CEFREM, the river discharges of ungauged catchments are estimated from a simple annual water balance model, which is greatly limited by meteorological forcing and the empirical description of hydrological processes. In ORCHIDEE, the water and energy exchanges between the surface and the atmosphere are described quantitatively with physically-based equations. It allows a better representation of the discharge of the river, which spatially integrates all upstream hydrological processes through high-resolution, river-routing parametrization^19^. Over unmonitored catchments, ORCHIDEE also benefits from data fusion since the correction factor is interpolated from neighbouring gauged rivers.

### The role of submarine groundwater discharge (SGD)

Another contributing factor to the 170–230 *km*^3^/*y* difference of freshwater inflow into the Mediterranean between FOG and previous studies is that the latter were mostly based on observed surface water, so they neglected SGD. This flux is represented implicitly by ORCHIDEE, because it fulfils the water continuity equation, and, thus, the moisture that converges over the continents (precipitation minus evaporation) will flow into the ocean at one point or another. The assimilation of the data corrects the errors in moisture convergence over the continents, and it is performed over large catchments that are not affected by SGD. The computed increments correspond to a moisture convergence correction, thereby improving the implicit representation of SGD in ORCHIDEE.

It has been reported that the submarine or coastal karst comprises 60% of the shoreline of the Mediterranean Sea^29^, and most of them are in Europe^30,31^. UNESCO^29^ mentioned that karstic systems account for around 75% of the freshwater input into the Mediterranean Sea with most of the flux being SGD. UNESCO^32^ and Zektser *et al*.^33^ estimated SGD freshwater flows into the Mediterranean Sea of 52 and 68 *km*^3^/*y*, respectively. Margat and Treyer^24^ obtained from national water statistics a total flux of 43 *km*^3^/*y* with Italy, Turkey and Croatia reporting the largest contributions. Rodellas *et al*.^34^ reported a value in the range of 300–4800 *km*^3^/*y* of submarine groundwater exchanges, of which 1–25% is fresh groundwater. The SGD estimate over the Black Sea is about 16 *km*^3^/*y* (lower than our error bar), which was obtained from tracer information (222Rn, salinity, and 18 O/2H) and satellite data (Sea Surface Temperature, Digital Elevation Model, fault system analysis)^35^.

Our result indicates that SGD has an important role in the water cycle of the Mediterranean Sea. CEFREM-HR probably is the most reliable estimate of total surface water inflow into the MED because it is based on the largest collection of observations of river discharges. Based on its difference to FOG, it can be inferred that SGD is probably smaller than 148 *km*^3^/*y* (i.e., FOG minus CEFREM-HR), and this flux is located mostly in the ALB, SWE, TYR, CEN, and ION sub-basins, where the discrepancies are largest. This is consistent with the fluxes reported by country^24^. In the arid and semi-arid countries of the Mediterranean region, SGDs are considered strategic freshwater resources^29^. SGDs are also important sources of nutrients, trace metals, and alkalinity to the Mediterranean System^34,36^, and so they are key factors in the eutrophication of coastal ecosystems.

## The Trend of Freshwater for the Period 1980–2008

### The climatic and non-climatic trend

The availability of the GRDC observations has decreased from about 80% of all stations in 1980 to less than 30% in 2008 (Fig. 2), thus the trend analysis is restricted to the period 1980–2008. During this period, the freshwater estimates obtained by FOG do not exhibit significant trends over most Mediterranean sub-basins, with the exceptions of ALB and ADR (Table 2). In these two sub-basins, a majority of ensemble members, which sample the uncertainty in FOG, display significant trends. It should be pointed out that the trend of freshwater depends strongly on the selected period. Milly *et al*.^37^ report decreased river discharges in souther European rivers during the 20th century. This is in agreement with the observed decreased from the 1960 s to the 1990 s (400 to 340 *km*^3^/*y*) documented by Ludwig *et al*.^14^. For a more recent period (1980 to 2000) no significant change is reported by Bouraoui *et al*.^8^ as we observe in the FOG dataset. Over the Black Sea, the trend in FOG is not significant, which is consistent with the results of Ludwig *et al*.^14^ over a longer period.

**Table 2:** Mean freshwater flux and its trend over the 11 sub-basins from 1980 to 2013.

Basin	Mean freshwater inflow [*km*^3^/*y*]	Total trend [*km*^3^/*y*/10*y*]	Climatic trend [*km*^3^/*y*/10*y*]	Non-climatic trend [*km*^3^/*y*/10*y*]
Alboran (ALB)	14 ± 4	**1.77** (**24/30)**	1.60	**0.18** (2/30)
South Western (SWE)	32 ± 20	0.80 (6/30)	0.75	0.05 (2/30)
North Western (NWE)	117 ± 51	−2.99 (6/30)	−4.46	1.37 (3/30)
Tyranian (TYR)	57 ± 11	−0.38 (0/30)	−0.33	−**0.05** (6/30)
Adriatic (ADR)	125 ± 31	−**8.02** (**19/30**)	−5.53	−2.49 (7/30)
Ionian (ION)	46 ± 5	1.09 (0/30)	−1.10	−0.01 **(20/30)**
Central (CEN)	28 ± 0.3	−0.24 (0/30)	−0.21	−**0.03** (3/30)
Agean (AEG)	70 ± 18	0.91 (0/30)	1.03	−**0.12 (30/30)**
North Levantine (NLE)	54 ± 3	−3.15 (2/30)	−0.99	−**2.17 (26/30)**
South Lenatine (SLE)	25 ± 6	−0.22 (0/30)	−0.195	−**0.02** (4/30)
Black Sea (BLS)	367 ± 61	0.56 (0/30)	3.88	−3.32 (7/30)

The underlined bold numbers indicate a significant trend in FOG at the 5% confidence level for the Mann-Kendall test. The numbers in parentheses indicate the numbers of the ensemble members in which a significant trend is detected. A bold font is used when FOG displays a significant trend or a majority of the members are in this case.

**Figure 4:**
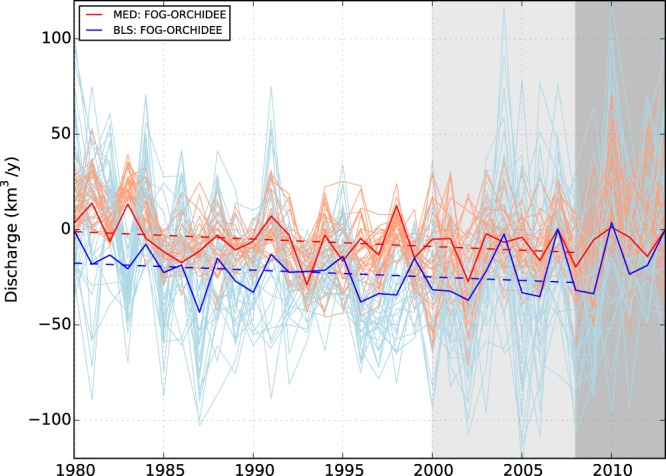
The non-climatic trend of river discharge into the Mediterranean Sea (red) and the Black sea (blue) together with the uncertainties (light colour lines) obtained from FOG minus ORCHIDEE over 1980–2008. The grey and dark grey shaded area indicates the period where the percentage of GRDC stations are lower than 40% and lower than 30%, respectively.

If we assume that time-dependent biases are negligible within ORCHIDEE, we can use the reference simulation (i.e., without data fusion) to estimate climate-driven trends in river discharges. We find that none of the sub-basins has a significant climatic trend for the 1980–2008 period (Table 2, Fig. 4). It is known that the MED has experienced decreasing precipitation during the 20th century^38^, but this does not necessarily lead to reduced streamflows as evaporation changes with climate as well. However, the non-climatic trends, which can be estimated by subtracting ORCHIDEE from the FOG ensemble, have significant values for a majority of members in several sub-basins (Table 2). The sub-basins with the most robust negative trends are in the Eastern Mediterranean, i.e., ION, AEG, and NLE, but, although significant, these trends, except for NLE, are an order of magnitude smaller than the climatic trends. The detection of climatic trends is limited by the magnitude of the variability of the climate, but non-climatic trends are tested against the error estimate, which is a weaker constraint. The decomposition of the total trends found in FOG also allows to emit the hypothesis that tendencies found for ALB and ADR are dominated by the climate signal since the non-climatic contribution is small (1/10th and 1/3rd, respectively).

### Possible factors that contributed to the significant non-climatic trend

Since the data fusion tends to decrease the riverine flow into the Mediterranean, it is unlikely that the decreasing number of stations in the GRDC database leads to the significant non-climatic trend that is identified. The reduced flow to the ocean in the eastern Mediterranean points to increased evaporation over the continents. Our result is consistent with the increased vegetation activity over the Balkan, southern Italy, and Turkey detected by Garcia-Ruiz *et al*.^3^ using the Normalized Difference Vegetation Index. The decreasing flows into the NLE and AEG can be explained by increases in irrigated areas and cultivation of more water intensive crops in Turkey and Greece^39,40^ which lead to higher evaporation and biomass production. For the NLE basin, these land and water usage changes seem to have a larger impact on the continental water cycle than the climatic trends over the period analysed.

## Conclusions

The proposed freshwater flux from the continents into the Mediterranean Sea, combines the quality of discharge observations with the predictive capabilities of an LSM. This estimate will help better constrain the water cycle of the Mediterranean Sea^6^ and improve our ability to predict the response of the Mediterranean Sea to the evolution of climate and to human water and land use. Since the intensification of agriculture began before the period considered here, it would be desirable to reconstruct freshwater fluxes into the sea over a longer period. This will be difficult because of the lower quality of atmospheric re-analyse (the basis for driving LSMs) before 1979 and the degrading density of the gauging station network available after 1990. This is a call to the community to come together to consolidate the observational databases and implement long-term re-analysis of the continental water cycle. In regions such as the Mediterranean, where renewable water resources are over exploited, long-term estimates of the continental water cycle, such as the one proposed here, are important tools because they allow fluctuations in resources to be attributed either to climate change or the evolution of the uses of water and land.

## Method

### The model and assimilation methodology

The ORCHIDEE land surface model (LSM)^18,19^ was used to simulate the continental water cycle over the period 1979–2013 using the WFDEI-GPCC atmospheric conditions^41^. These conditions were derived from the ERA-Interim re-analysis^42^ and bias corrected with *in-situ* observations. In particular, precipitation was adjusted with the Global Precipitation Climatology Centre’s (GPCC’s) dataset^43^. Using the methodology described in Wang *et al*.^21^, the observations of rivers’ discharges collected by the Global Runoff Data Center (GRDC), 56068 Koblenz, Germany were assimilated to correct the simulated runoff and drainage, or equivalently, the moisture convergence (precipitation minus evaporation), as the fusion preserves the water conservation of the model. The resulting correction factor (x), which optimises the freshwater discharge into the sea, only applies upstream of the gauging station. In order also to correct the water cycle of unmonitored basins, x was extrapolated to the entire drainage basin of the Mediterranean using a simple linear interpolation. The estimated flows of freshwater into the Mediterranean obtained through this fusion of ORCHIDEE and GRDC are labelled “FOG”.

The ORCHIDEE simulation uses a constant vegetation distribution map to avoid the influence of non-climatic drivers on discharge fluctuations. The Nile river has a catchment area of 2,893,410 *km*^2^, but it only contributes 14 *km*^3^/*y* (during 1984–1985) of freshwater to the Mediterranean Sea^44^. These low values result from the large inner delta and marshlands between lake Victoria and the Sahara as well as the intense water use for irrigation allowed by the Aswan Dam and other infrastructures in Egypt^14,20,24^. In order to reduce the computational cost of ORCHIDEE and the assimilation method, the catchment of the Nile was excluded from the domain and its discharge set to 6.1 *km*^3^/*y*. The value was obtained by averaging the observations at the El Ekhsase station over the 1980–2009 period. In our review of previous MCB estimates of the freshwater flux into the MED, we have only kept those values where we could subtract their Nile discharge.

### Error modelling

To provide a measure of the uncertainty of FOG, an error model was developed for the model/data fusion method used here. Comparing the optimal solution to the observations used in the assimilation allowed us to determine the variance of the residual error in FOG. For periods during which no observations were available and, thus, climatology was assimilated, the inter-annual variance was used to characterise the error. To explore the uncertainty of FOG, the error variance defined above was used to generate annual perturbations to the correction factor, x, assuming a normal distribution. Running ORCHIDEE with 30 of the perturbed corrections allowed us to predict as many discharges into the Mediterranean that are within the residual error or inter-annual variability, depending on the information used for FOG. The uncertainty of FOG was estimated using the 95% confidence interval of the 30 ensemble members and it is essentially driven by the availability of GRDC stations (Fig. S2). This ensemble of solutions was the basis for the statistical evaluation of the results.

### The dataset

Of the 792 GRDC stations over the domain that was studied (19.7°W-62.7°E, 25°N-62°N, Fig. 1) only 338 of the stations reported observations over the period of simulation (1979–2013) and could be placed in the modelled catchments while allowing some margin of error for the position and upstream area. Eighty-eight GRDC stations are located within the catchment of the Black Sea. For the catchments of the ALB, SWE, TYR, CEN, ION, NLE, and SLE oceanic basins (Fig. 1), less than 10 stations are available. The GRDC stations outside of the Mediterranean catchment also contribute to constraining the water cycle over the region through the interpolation of the correction factor. The CEFREM only uses the GRDC stations closest to the coastal points to obtain an estimate at the river outlet. CEFREM-LR (HR) uses observations for only 1.3% (0.2%) of the coastal points, but the rivers that lead there cover 65.0% (77.4%) of the total catchment. The estimated discharge over unobserved catchments was obtained from an annual water balance between evaporation and precipitation^7,14^. The CEFREM data were available at both High Resolution (CEFREM-HR, 0.083°, 1980–2009) and Low Resolution (CEFREM-LR, 0.5°, 1960–2000). They differ by the number of gauging stations used and the atmospheric forcing. The CEFREM-LR was the basis for the publication by Ludwig *et al*.^14^, while the CEFREM-HR is an updated version that was obtained from the authors in 2015 (Ludwig personal communication, 2015).

### Estimation of the climatic and non-climatic trends of FOG

The freshwater flux estimated by ORCHIDEE only includes climate fluctuations and it has been shown that the LSM approach to estimating moisture convergence trends is currently the most reliable method^45^. FOG adds a correction to this climate variability that combines the time invariant bias of the LSM, non-climatic changes in the water cycle, and time dependent error in the LSM and atmospheric forcing. Thus, the trends in the difference between these two estimates can be attributed only to climate independent changes in evaporation through water usage, the evolution of vegetation, time-dependent errors in the model. We assumed here that this last component did not contribute any systematic trends.

## Supplementary information

Supplementarial Material

## Data Availability

The FOG data are freely available on the HyMeX database: http://mistrals.sedoo.fr/?editDatsId=1500&datsId=1500. The related source code is available from the authors on request.
