# Supplementary material for: Assessing the freshwater flux from the continents to the Mediterranean Sea
Source: Sci Rep. 2019 May 29;9:8024. doi: 10.1038/s41598-019-44293-1 (PMC6541616; doi:10.1038/s41598-019-44293-1)
Supplement: Supplementary file 1 — Supplementarial Material [file 41598_2019_44293_MOESM1_ESM.pdf]

Assessing the freshwater flux from the continents to the Mediterranean sea : supplementary information

Fuxing Wang[1], Jan Polcher[1]

[1]*Laboratoire de Météorologie Dynamique, IPSL, CNRS, Ecole Polytechnique, 91128, Palaiseau, France*

List of Figures

S1 The colour scale illustrates the difference in discharge by sub-basins between FOG and CEFREM-HR. The percentage for each sub-basin provide the range of the corresponding relative differences as derived from 95% confidence interval of the FOG ensemble. 3

S2 Relation between the number of GRDC stations available each year over the 1980-2013 period and the estimated uncertainty for the total discharge into the MED and BLS. 4

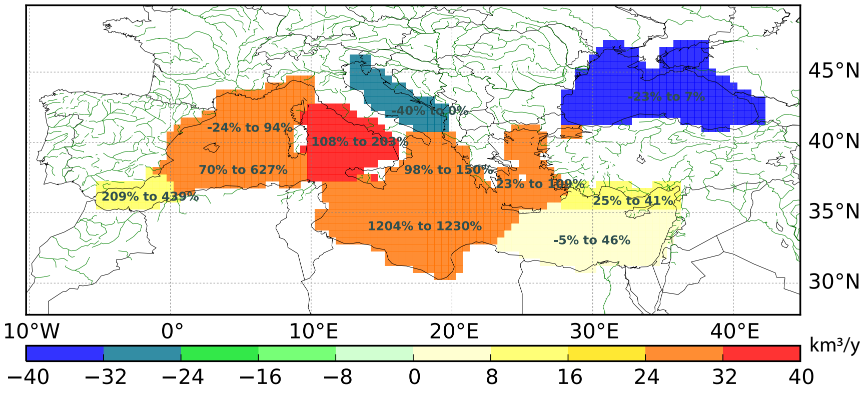

Figure S1: The colour scale illustrates the difference in discharge by sub-basins between FOG and CEFREM-HR. The percentage for each sub-basin provide the range of the corresponding relative differences as derived from 95% confidence interval of the FOG ensemble.

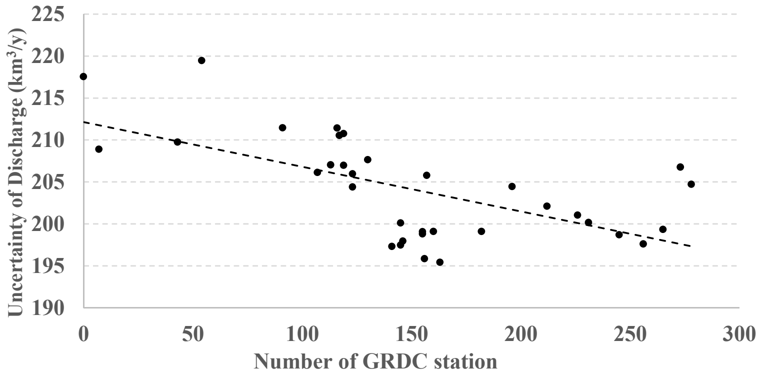

Figure S2: Relation between the number of GRDC stations available each year over the 1980-2013 period and the estimated uncertainty for the total discharge into the MED and BLS.
